# Fibroblasts from phenotypically normal palmar fascia exhibit molecular profiles highly similar to fibroblasts from active disease in Dupuytren's Contracture

**DOI:** 10.1186/1755-8794-5-15

**Published:** 2012-05-04

**Authors:** Latha Satish, William A LaFramboise, Sandra Johnson, Linda Vi, Anna Njarlangattil, Christina Raykha, John Michael Krill-Burger, Phillip H Gallo, David B O'Gorman, Bing Siang Gan, Mark E Baratz, Garth D Ehrlich, Sandeep Kathju

**Affiliations:** 1 Department of Surgery, Division of Plastic Surgery, University of Pittsburgh Medical Center, Pittsburgh, PA, USA; 2 Department of Pathology, University of Pittsburgh, Pittsburgh, PA, USA; 3 Center for Genomic Sciences, Allegheny-Singer Research Institute, Allegheny General Hospital, Pittsburgh, PA, USA; 4 Cell and Molecular Biology Laboratory of the Hand and Upper Limb Centre, St. Joseph's Hospital, London, ON, Canada; 5 Division of Upper Extremity Surgery, Department of Orthopaedics, Allegheny General Hospital, Pittsburgh, PA, USA

## Abstract

**Background:**

Dupuytren's contracture (DC) is a fibroproliferative disorder characterized by the progressive development of a scar-like collagen-rich cord that affects the palmar fascia of the hand and leads to digital flexion contractures. DC is most commonly treated by surgical resection of the diseased tissue, but has a high reported recurrence rate ranging from 27% to 80%. We sought to determine if the transcriptomic profiles of fibroblasts derived from DC-affected palmar fascia, adjacent phenotypically normal palmar fascia, and non-DC palmar fascial tissues might provide mechanistic clues to understanding the puzzle of disease predisposition and recurrence in DC.

**Methods:**

To achieve this, total RNA was obtained from fibroblasts derived from primary DC-affected palmar fascia, patient-matched unaffected palmar fascia, and palmar fascia from non-DC patients undergoing carpal tunnel release (6 patients in each group). These cells were grown on a type-1 collagen substrate (to better mimic their *in vivo *environments). Microarray analyses were subsequently performed using Illumina BeadChip arrays to compare the transcriptomic profiles of these three cell populations. Data were analyzed using Significance Analysis of Microarrays (SAM v3.02), hierarchical clustering, concordance mapping and Venn diagram.

**Results:**

We found that the transcriptomic profiles of DC-disease fibroblasts and fibroblasts from unaffected fascia of DC patients exhibited a much greater overlap than fibroblasts derived from the palmar fascia of patients undergoing carpal tunnel release. Quantitative real time RT-PCR confirmed the differential expression of select genes validating the microarray data analyses. These data are consistent with the hypothesis that predisposition and recurrence in DC may stem, at least in part, from intrinsic similarities in the basal gene expression of diseased and phenotypically unaffected palmar fascia fibroblasts. These data also demonstrate that a collagen-rich environment differentially alters gene expression in these cells. In addition, Ingenuity pathway analysis of the specific biological pathways that differentiate DC-derived cells from carpal tunnel-derived cells has identified the potential involvement of microRNAs in this fibroproliferative disorder.

**Conclusions:**

These data show that the transcriptomic profiles of DC-disease fibroblasts and fibroblasts from unaffected palmar fascia in DC patients are highly similar, and differ significantly from the transcriptomic profiles of fibroblasts from the palmar fascia of patients undergoing carpal tunnel release.

## Background

Dupuytren's contracture (DC) is characterized by abnormal thickening of palmar fascia into collagen-rich cords that cause the fingers to bend and curl into a flexed and contracted state [1]. Although this disease can occur in both sexes, it is more common in men of Northern European descent [2-4] and typically presents in the 4th to 6th decade of life. DC has been reported to behave as a heritable genetic disorder, with evidence that it arises (in at least some cases) from an autosomal dominant gene on chromosome 16 with variable penetrance [3]. Lifestyle factors including smoking or heavy drinking [5,6], and heavy manual labor and hand trauma, have also been linked to development of DC [7,8], as have diabetes, epilepsy and hypercholesterolemia [9-11].

Treatment of DC remains problematic. A variety of non-surgical interventions, including injection of steroids [12] or gamma-interferon [13], use of creams based on vitamin E [14], dimethyl sulphoxides [15], and ultrasound therapy [16] etc. yield limited benefits. Recently, direct injection of clostridial collagenase has been evaluated with some promising results [17-19]. However, surgical excision of the involved contracted tissue remains the mainstay of therapy, supplemented with post-operative splinting and physical therapy [20,21]. Alternative therapeutic approaches remain desirable since surgery carries significant risks, including damage to the digital nerves and blood vessels, damage to the underlying flexor tendons, and wound healing failure with the possibility of skin necrosis.

A particularly vexing feature of DC is its propensity for recurrence despite the appearance of successful initial treatment. Many patients eventually require multiple surgeries with a cumulative risk of morbidity. However, it remains unclear what factors are responsible for recurrence of the disease. Apart from the possibility of a genetic predisposition, it has been hypothesized that undetected residual foci of incipient disease are present in the otherwise normal appearing and uninvolved palmar fascia, and that these cells represent sites of disease recurrence.

We have previously investigated the transcriptomic differences between fibroblasts derived from diseased DC cords versus fibroblasts from phenotypically normal palmar fascia in patients undergoing carpal tunnel (CT) release. These studies demonstrated intrinsic differences in gene expression between these cell populations that persisted even after propagation under cell culture. We have now extended these studies to include fibroblasts from macroscopically uninvolved (i.e. phenotypically normal) palmar fascia surgically removed from patients with DC. These fibroblasts, as well as fibroblasts from diseased DC cords and control carpal tunnel fibroblasts, were cultivated in cell culture on a type-1 collagen substrate to better approximate the *in vivo *collagen-enriched environment that these cells experience. The transcriptomic signatures of these three cell types were then compared to answer the question: do fibroblasts from phenotypically normal palmar fascia in DC more closely resemble their counterparts in phenotypically dissimilar DC cords, or cells from phenotypically similar carpal tunnel fascia?

## Methods

### Clinical specimens

Dupuytren's contracture (DC) cord samples and small samples of phenotypically normal palmar fascia tissue (PF) were surgically explanted at the Hand and Upper Limb Centre at St Joseph's Health Care (SJHC), London, ON, Canada. We also obtained phenotypically normal palmar fascia from patients undergoing carpal tunnel release (CT) in London, ON, Canada, and at the Allegheny General Hospital, Pittsburgh, PA. The study protocol conformed to the ethical guidelines of the 1975 Declaration of Helsinki. All specimens were collected under Institutional Review Board approval. Six patient samples of each tissue type were used to derive fibroblasts for use in these studies.

### Primary cell culture

Primary cultures of fibroblasts were purified from the surgically resected DC cord and matching specimens of normal appearing palmar fascia (PF), and from specimens of normal palmar fascia of patients undergoing carpal tunnel surgery (CT) as previously described [22,23]. The cultures were maintained in α-MEM-medium supplemented with 10% fetal bovine serum (FBS, Invitrogen Corporation, Carlsbad, CA) and 1% antibiotic-antimycotic solution (Sigma-Aldrich, St Louis, MO). All cultures were harvested prior to the sixth passage, with no changes in cell morphology observed during the *in vitro *expansion protocol. For the present study we used 6 primary DC and patient-matched PF cell cultures as well as primary fibroblasts purified and expanded from 6 patients undergoing surgical carpal tunnel release. Primary cultures of fibroblasts were grown on collagen monolayers as described previously [24]. In brief, collagen was coated on 6-well tissue culture plates with each well containing 880 μl of rat tail (type-1) collagen and 200 μl of the neutralization solution (2 parts 0.34N NaOH and 3 parts 10x Waymouth media) to a final concentration of 1.9 mg/ml. Following collagen polymerization, primary cultures were grown in α-MEM-medium containing 10% FBS and 1% antibiotic-antimycotic solution and the cultures were left undisturbed at 37°C at 5% CO_2 _for 72 hours.

### Total RNA extraction

Each well containing cells grown on collagen was treated with 1 ml of 0.25 mg/ml of collagenase XI (Sigma-Aldrich) at 37°C with gentle rotation for 20 mins, until the cells detached from the underlying collagen substrate. The samples were collected and centrifuged for 4 minutes at 900 rpm to concentrate the cells into a pellet for RNA purification. Total RNA was extracted using the RNeasy Mini Kit (Qiagen Inc., Valencia, CA) according to the manufacturer's instructions. The concentration of the extracted RNA was quantified using a Nanodrop ND-1000 Spectrophotometer (NanoDrop, Wilmington, DE) (absorption ratio 260/280 > 1.8). The samples were then analyzed on an Agilent 2100 Bioanalyzer (Agilent Technologies, Santa Clara, CA) to ensure adequate sample quality and absence of RNA degradation (RIN value > 8.0).

### Microarray assays

Total RNA (1.5 μg/sample) purified from fibroblasts derived from DC cord (n = 6), PF (n = 6) and CT (n = 6) specimens was subjected to analysis with Human WG-6 v3.0 Expression BeadChips (Illumina, SanDiego, CA), each of which contains six arrays on a single BeadChip. Each array is comprised of >48,000 probes derived from human genes in the NCBI RefSeq and UniGene databases. Each array on the BeadChip thus provides genome-wide transcriptional representation of well-characterized genes, gene candidates, and splice variants. A total of 18 separate samples were interrogated on the BeadChip arrays, i.e., there was no pooling of samples within groups. This allowed us to evaluate sample to sample variability in gene expression both within and across each group.

Total RNA was amplified using the Illumina^® ^TotalPrep™RNA Amplification Kit (Ambion, Austin, TX). Labeling and hybridization was performed according to the Illumina gene expression protocol, featuring a reverse transcription step to synthesize first strand cDNA followed by addition of second strand master mix containing DNA polymerase for second strand cDNA synthesis. This was followed by a single *in vitro *transcription (IVT) amplification step that incorporated biotin-labeled nucleotides. Subsequent steps included array hybridization, washing, blocking, and streptavidin-Cy3 staining followed by serial non-stringent washing steps to remove unbound conjugate. Following the final rinse, the bioarrays were dried by centrifugation and scanned. The Illumina bioarrays were read in an Illumina BeadArray Reader and the primary intensity data were obtained in standard file format, using Genome Studio software.

### Microarray expression analysis

The data obtained from the three cell sources were analyzed for differences in expression as previously described using the multiclass analysis tool of the Significance Analysis of Microarrays program (SAM version 3.02) [23,25]. We then performed unsupervised hierarchical clustering, concordance mapping and created Venn diagrams using the Partek Genomics Suite version 6.4 (St. Louis, MO) to evaluate similarities and differences in the gene expression patterns among the fibroblasts from each of the three cell sources.

### Pathway analysis

Statistically significant and differentially expressed genes determined by SAM were subjected to systematic network analysis to determine the primary biological processes and pathways associated with each group using "Ingenuity Pathways Analysis" (IPA, ver.5.0, Ingenuity^® ^Systems, http://www.ingenuity.com, Mountain View, CA). Only genes that were significantly increased or decreased were included as a defined parameter for the core analysis. Using information stored in the Ingenuity Pathways Knowledge Base (IPKB), genes that mapped to genetic networks were ranked by a score based on the number of eligible molecules contained in the network, which also defines the probability that each network can be achieved by chance alone. A score of 3 is considered threshold of significance which means that there is a 1 in 1000 chance that genes are integrated in the network by pure chance. Canonical pathways were also identified from the IPA library. The significance of the association between the data set and the canonical pathway was measured in two ways within the Ingenuity program: i) as a ratio of the number of genes from the data set that map to the pathway divided by the total number of genes that map to the canonical pathway was calculated, and ii) by the Fischer's exact test which was used to calculate a *p*-value determining the probability that the association between dataset genes and the canonical pathway is significant.

### Quantitative real time RT-PCR

The protocol for reverse transcription reactions and real time PCR was followed as previously described [23]. Ninety ng of total RNA and 150 ng of random primers (Invitrogen Corporation) were used in 30 μL reactions and real time RT-PCR was performed with triplicate sample reads. The 18 samples were prepared separately such that gene expression analysis was performed using individual samples without pooling. Real time PCR amplification and detection of template was carried out using Applied Biosystems transcript-specific assays including: angiopoietin-like 7 (ANGPTL7) - Hs00221727_m1, Laminin alpha 5(LAMA5) - Hs00966585_m1, Shroom 2 - Hs01113636_m1, and GAPDH - Hs02758991_g1. Using the comparative critical cycle (Ct) method and using GAPDH as the endogenous control, the expression levels of the target genes were normalized using a 95% confidence interval. The relative expression of the 18 samples (6 for each subset) was averaged and statistical analysis for significance was performed using a Student's *t*-test. Results shown are from two independent experiments performed in triplicate.

## Results

**Figure 1:**
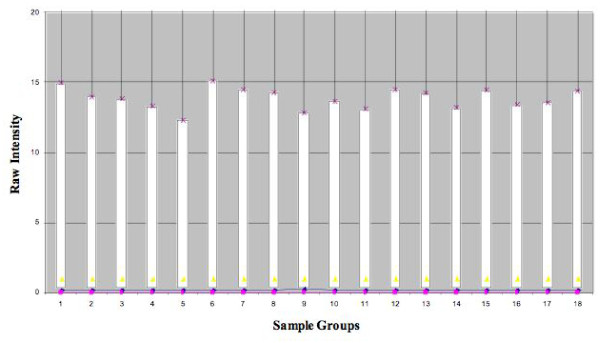
**Box Plot Analysis of the Three Sample Groups**. Box plot analysis of the distribution of the intensity values across the 3 groups demonstrated comparable dispersion of the individual data sets regardless of cell source, eliminating the need for normalization or smoothing of the raw data. 1-6: palmar fascia-derived fibroblasts (PF). 7-12: Dupuytren's contracture-derived fibroblasts (DC). 13-18: carpal tunnel -derived fibroblasts (CT)

The raw intensity values representing expression of individual transcripts were corrected by subtraction of the background intensity for each array. All values that were at or below background concomitantly in all 3 sample groups were removed yielding 27214 transcripts for further analysis. Box plot analysis of the distribution of these intensity values across the three groups demonstrated comparable dispersion of the individual data sets regardless of patient source, eliminating the need for log normalization or secondary smoothing of the raw intensity data prior to statistical evaluation (Figure 1).

Transcript intensity values were analyzed for differences in individual gene expression levels using the multiclass comparison of the SAM program (Tusher et al., 2001). The overall raw data distribution was not modified prior to analysis except for median centering of the array data within the SAM program. Results were considered significant at a false detection rate (FDR) of 5% (q ≤ 0.05) and the multiclass comparison revealed 959 transcripts differentially expressed among the 3 classes (delta = 0.47 and FDR = 4.89%). Removal of cDNA clones, open reading frame transcripts, redundant probes and transcripts for hypothetical proteins among the 959 significant transcripts revealed 724 distinct, functionally annotated gene products that were differentially expressed among the 3 groups (Additional file 1).

**Figure 2:**
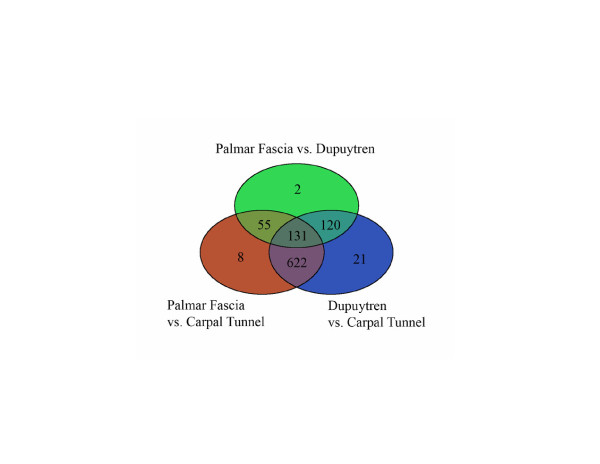
**Venn Diagram of Individual Group Comparisons Among the Three Sample Groups**. Each of the circles depicts the number of different transcripts based on a binary comparison for each of the labeled sample groups (green, orange, blue) from among the 959 transcripts identified as statistically significant in the study (SAM). Overlapping differences shared among more than one sample group comparison are represented in the areas of intersection between 2 circles. The 131 transcripts in the center of the Venn Diagram represents genes that are different among all group comparisons. The number of transcripts in the carpal tunnel comparisons (622) far exceeded those in the palmar fascia (55) and Dupuytren (120) comparisons

Post hoc comparisons were performed on these 959 transcripts against the Student's t-distribution using the Partek Software for paired (Dupuytren samples: DC and PF) and the unpaired samples obtained during carpal tunnel release (CT). Three separate univariate comparisons resulted in 894 transcripts detected as significantly different in DC vs CT-derived fibroblasts, 816 significantly different transcripts between PF and CT-derived fibroblasts, and 308 differentially expressed transcripts between DC and PF-derived fibroblasts. These lists are depicted in a Venn Diagram (Figure 2) that illustrates the significant differences specific to each set of individual comparisons (within a circle) versus those shared in more than one of the statistical comparisons (shared between circles). For example, the comparison of DC vs CT-derived fibroblasts yielded 21 transcripts in the non-overlapping area (blue) that were different between these cell types but were not significantly different in the other two comparisons. In contrast, the overlapping region between the PF versus DC circle (green) and the DC vs CT circle (blue) contained 120 transcripts. These 120 transcripts were significantly different in comparisons of DC to either PF or CT-derived fibroblasts but were not different between PF and CT-derived fibroblasts (orange). There were 131 transcripts in the center of the Venn Diagram that overlapped among all three circles, indicating that these transcripts were significantly different in all three comparisons. The most interesting observation, however, is that 622 transcripts (pink) were significantly different in comparisons of CT- to both PF- and DC-derived fibroblasts, but were similarly expressed in DC and PF cells.

**Figure 3:**
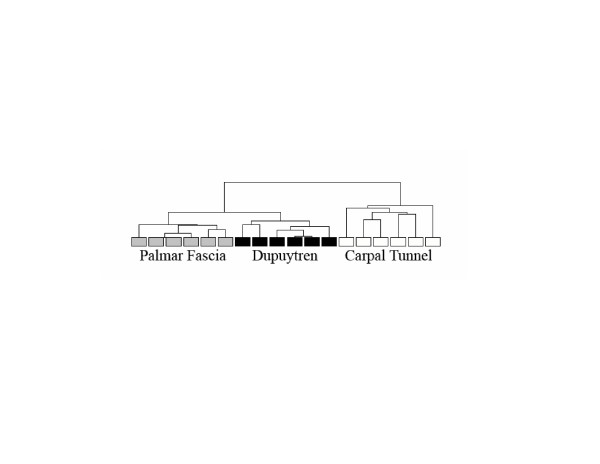
**Unsupervised Hierarchical Clustering of Expression Values Based on Sample Origin**. The sample distributions of significantly different transcripts (959 transcripts, SAM v. 3.02) are displayed based on unsupervised, hierarchical, agglomerative clustering (Partek: v. 6.4) of log base 2 intensity values. A primary branch of the dendrogram distinguishes the expression values obtained from transcripts of the palmar fascia- and Dupuytren's-derived fibroblasts from those of the carpal tunnel-derived fibroblasts, indicating a distinct difference based on these tissues of origin. Dupuytren's and palmar fascia-derived fibroblasts also formed distinct clusters separate from each other at the next branching level despite their matched patient origin

Unsupervised hierarchical clustering was performed using the Partek program on log base 2 intensity values of the differentially expressed transcripts identified by the SAM program. Agglomerative clustering was performed to detect dissimilarity based on Euclidean distance with clusters linked based on average values in order to determine if major variations existed in gene expression profiles among the 3 groups [26]. The program was set to delineate the maximum number of clusters based on individual sample comparisons. The primary branch of the resulting dendrogram separated the transcripts of the PF and DC-derived fibroblasts from those of the CT-derived fibroblasts, indicating that significant differences in expression were based on patient origin (Figure 3). However, the DC- and PF-derived fibroblasts also formed clearly distinct clusters separate from each other at the next level of branching. Thus, all three cell groups exhibited distinctly different expression signatures with the greatest difference between the unpaired and the paired (DC-derived) samples.

**Figure 4:**
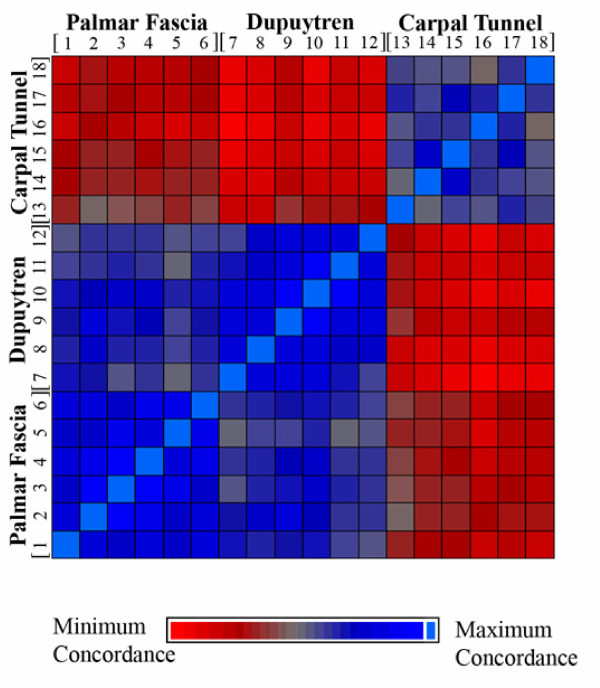
**Concordance Analysis of Individual Sample Profiles of Gene Expression**. Concordance was calculated (Partek) using the initial dataset of 959 significant transcripts (log base 2) with similarity between samples derived through Euclidean distance based on Euclidian distance. Relative similarity among samples based on labeled origin is depicted through a color gradient where dark blue represents highest similarity of expression values on the scale diminishing across a gradient to the most different values represented in dark red color. The light blue color at the blue-red interface is indicative of sample identity. Based on this analysis, palmar fascia- [1-6] versus Dupuytren-derived fibroblast samples [7-12] exhibited highest similarity (blue), while both were substantially different from carpal tunnel-derived fibroblast [13-18] expression profiles

Concordance analysis was performed to delineate similarities in expression profiles among samples. Concordance was calculated using the initial dataset of 959 significant transcripts (log base 2) with similarity between samples based on Euclidean distance (Figure 4). Relative similarity is depicted by a color gradient where blue represents the highest similarity diminishing to the most different values represented in red color. The light blue color at the blue-red interface was indicative of sample identity. Based on this analysis, expression of PF- [1-6] and DC-derived fibroblasts [7-12] exhibited the highest similarity (blue) while both were markedly different from CT-derived fibroblast [13-18] profiles, consistent with the results obtained via hierarchical clustering.

### Identification of biological pathways

Biological interactions among genes differentially expressed between Dupuytren's-derived and CT-derived fibroblasts were identified using Ingenuity^® ^System Analysis. Two distinct gene sets of interest were analyzed: 1) genes concordantly expressed between PF- and CT-derived fibroblasts but significantly different in DC-fibroblasts (120 genes); and 2) genes concordantly expressed between DC- and PF-fibroblasts but significantly different in CT (622 genes). The program identified 19 and 172 genes respectively that were eligible for function/pathway analysis, comprising 2 and 12 networks respectively with scores of 3 or greater (Tables 1 and 2). The 19 eligible genes identified from the first gene set represented functional categories such as cell death, cell cycle, cellular growth and proliferation- all understandably relevant to a fibroproliferative process like Dupuytren's (Table 1).

**Table 1:** Ingenuity analysis of genes concordantly expressed between PF- and CT-derived fibroblasts but significantly different in DC-fibroblasts

ID	Molecules in Network	Score	Focus Molecules	Top Functions	Canonical Pathways
1	ARID4B, ATRX, C1ORF25, CCL7, CDKN1A, CEP350, EGLN1, EPOR, FAM124B, FBXO9, GRIN1, HDAC1, HNF1A, IMPA2, KCNE4, MIR106A (includes EG:406899), MIR17 (includes EG:406952), MIR20B (includes EG:574032), MXI1, MYC, MYCT1, NFIA, NFkB (complex), NRG1, PPARA, RORA, SAP130, SEMA4B, Sin3A, SKP1, SUDS3, TNF, TRIP6, ZNF442, ZNF33B	53	18	Cell Death, Cell Cycle, Cellular Growth and Proliferation	DNA Methylation and Transcriptional Repression Signaling, Toll-like Receptor Signaling, TREM1 Signaling. Erythropoietin Signaling, PXR/RXR Activation, LXR/RXR activation, Melatonin Signaling, Chemokine Signaling, PPAR Signaling, FXR/RXR Activation, HIF1∝ Signaling, Hepatic Cholestasis, Aryl Hydrocarbon Receptor Signaling, Inositol Phosphate Metabolism, PPAR∝/RXR∝ Activation, Production of Nitric oxide and Reactive Oxygen Species in Macrophages, LPS/IL-1 Mediated Inhibition of RXR Function, Axonal Guidance Signaling
2	HSPB3, LSM14B	3	1	Cellular Compromise, Cellular Function and Maintenance	

**Table 2:** Ingenuity analysis of genes concordantly expressed between DC- and PF-fibroblasts but significantly different in CT

ID	Molecules	Score	Focus Molecules	Top Functions	Canonical Pathways
1	ABCG5, Ap1, BIRC3, CASP1, Caspase, CCR6, Creb, CSF2RA (includes EG:1438), DAPP1, DDOST, DMC1, ERK1/2, HNRNPU, IL1, IL18, IL12 (complex), IL17RD, Jnk, LEP, MECOM, NAPB, NFkB (complex), PDGF BB, PNPT1, RAD51, RNF31, SLC12A4, SSTR2, STAT5a/b, SUMO1, TNFSF14, TNFSF15, TP53BP2, UNC5B, XRCC2	47	25	Cell Cycle, DNA Replication, Recombination, and Repair, Metabolic Disease	Pyrimidine Metabolism, Artherosclerosis Signaling, TREM1 Signaling, Glycosphingolipid Biosynthesis-Lactoseries, Death Receptor Signaling, Nucleotide Sugars Metabolism, Aminosugars Metabolism, LXR/RXR Activation, Axonal Guidance Signaling, FXR/RXR Activation, Amyotrophic Lateral Sclerosis Signaling, Endothelin-1 Signaling, N-Glycan Degradation, Cholecystokinin/Gastrin-mediated Signaling, p38 MAPK Signaling, Glycosaminoglycan Degradation, T Helper Cell Differentiation, Graft-versus-Host Disease Signaling, Ovarian Cancer Signaling, Chondroitin Sulfate Biosynthesis, Role of Cytokines in Mediating Communication between Immune Cells, Lymphotoxin β Receptor Signaling, Role of BRCA1 in DNA Damage Response, Dendritic Cell Maturation, Induction of Apoptosis by HIV1, IL-10 Signaling, JAK/STAT Signaling, GM-CSF Signaling, Hypoxia Signaling in the Cardiovascular System, Communication between Innate and Adaptive Immune Cells, Antiproliferative Role of Somatostatin Receptor 2, Linoleic Acid Metabolism, Acute Phase Response Signaling, Acute Myeloid Leukemia Signaling, leptin Signaling in Obesity, Altered T Cell and B Cell Signaling in Rheumatoid Arthritis, Role of Pattern Recognition Receptors in Recognition of Bacteria and Viruses, PPAR Signaling, Apoptosis Signaling, IL-6 Signaling, Crosstalk between Dendritic Cells and Natural Killer Cells, p53 Signaling, Chronic Myeloid Leukemia Signaling,
2	ACTR3, ACVR2A, ADCY, AHR, Akt, CAPZA1, CHRNA5, Ck2, CTDP1, ERK, FLNC, FSH, ganglioside GD1b, GATA6, hCG, Histone h3, Histone h4, HSD17B7, Lh, MAGT1, Mapk, MIR124, NELF, NME2, P38 MAPK, PI3K, Pka, PPIB, PROS1, RNA polymerase II, SNAPC4, STAR, STMN1, UPP1, USP49	33	19	DNA Replication, Recombination, and Repair, Drug Metabolism, Endocrine System Development and Function	Pyrimidine Metabolism, Purine Metabolism, N-Glycan Biosynthesis, Axonal Guidance Signaling, Androgen and Estrogen Metabolism, Factors Promoting Cardiogenesis in Vertebrates, Coagulation System, RhoA Signaling, Role of NANOG in Mammalian Embryonic Stem Cell Pluripotency, AMPK Signaling, Estrogen-Dependent Breast Signaling, Activation of IRF by Cytosolic Pattern Recognition Receptors, Wnt/β-catenin Signaling, Caveolar-mediated Endocytosis Signaling, Ephrin Recpetor Signaling, NRF2-mediated Oxidative Stress Response, TGF-β Signaling, Protein Ubiquitination Pathway, Thrombin Signaling, Regulation of Actin-based Motility by Rho, Virus Entry via Endocytic Pathways, Fcy Receptor-mediated Phagocytosis in Macrophages and Monocytes
3	C10ORF58, C6ORF170, CCNB2, CDC7, CPSF4, FBXL12, FCAR, FKBP11, GADD45B, IL9, IL24, Il12 receptor, ITIH3, ITIH5, ITPA, KIAA0101, MAN2B2 (includes EG:23324), MARK3, MCART1, NFYB, PCNA, PFN2, PGRMC1, PILRA, POLD1, PTGS1, RORA, RRAD, STAT4, TBX21, TERT, TGFB1, TGFBR2, XBP1, ZNF394	22	14	Cellular Development, Cell Morphology, Cell-Mediated Immune Response	Pyrimidine Metabolism, Purine Metabolism, Cleavage and Polyadenylation of Pre-mRNA, Eicosanoid Signaling, Endothelin-1 Signaling, N-Glycan Degradation, Arachidonic Acid Metabolism, Ovarian Cancer Signaling,
4	Amino acids, BACE1, BLZF1, C1D, CBR3, CCAR1, CDC6, COL4A3BP, CSNK1G2, EXO1, FEN1, GSTA5, HNF4A, MCM4, MCM5, MCM6, MCM8, MEG3 (includes EG:55384), MLH1, MST1, PMM1, PPARG, PPP1R15B, PRKY, PTGR2, RAB2A, RORC, SAP30BP, SFRS11, SORBS1, STK16, TEAD3, TP53, VDAC2, XPNPEP3	21	14	Tumor Morphology, Cancer, Cell Cycle	Wnt/β-catenin Signaling, Inositol Metabolism,
5	C12ORF24, C3ORF34, C4ORF43, CBR3, CDKN2AIPNL, CEBPB, CYP1A1, D-glucose, DAG1, DCAF13, ETFDH, F7, FKTN, HNF1A, HNF4A, IL1B, INS, LRSAM1, MAPK8, NCK1, NFkB (complex), PHF23, POFUT1, PRR3, SESN2, SLC25A32, SP1, SRC, TSG101, UBE2D3 (includes EG:7323), UBE2N, ZNF557, ZNF577	20	13	Carbohydrate Metabolism, Molecular Transport, Small Molecule Biochemistry	
6	ATP, BACE1, C14ORF153, CASP3, CRCP, CTSD, EEF2, EIF2AK3, ERBB2, EREG, ERP29, FUT3, GALNT3, ganglioside GD1a, GHR, GRIPAP1, HIVEP2, HLA-DRA, HRAS, IL24, MSH2, MYO9B, N4BP2, NLRP3, ODZ3, PPP1R15A (includes EG:23645), PRTN3, RFX4, RFXANK, Rsk, SERPINE2, Shc, Sod, SULT1A1, TNF	19	13	Cellular Assembly and Organization, Tissue Morphology, Cell Death	Pyrimidine Metabolism, Purine Metabolism, Cysteine Metabolism, Glycosphingolipid Biosynthesis-Lactoseries, Sulfur Metabolism, Glycosphingolipid Biosynthesis-Neolactoseries, O-Glycan Biosynthesis, Keratan Sulfate Biosynthesis, Chondroitin Sulfate Biosynthesis, BMP Signaling Pathway, NRF2-mediated Oxidative Stress Response, EIF2 Signaling
7	C9ORF80, CDC45L, CTSD, DEFB1, EEF2, Eotaxin, ERAP2, GADD45B, GHR, GRM8, HMGA1, IFNB1, IFNG, IRF9, LILRB3 (includes EG:11025), LIMK1, LMNB2, MSH2, MSH3, MYC, ORC1L, ORC2L, ORC6L, PHC2, PNPT1, RHOB, Rock, ROCK2, RPL7, SEPHS2, SERPING1, STK11, TLE1, TMEM70, UTY	18	12	Cell Cycle, Gene Expression, Inflammatory Response	Pyrimidine Metabolism, Nucleotide Sugars Metabolism, Aminosugars Metabolism, Axonal Guidance Signaling, Cholecystokinin/gastrin-mediated Signaling, Complement System, RhoA Signaling, Glycosaminoglycan Degradation, CCR3 Signaling in Eosinophils, Selenoamino Acid Metabolism, Semaphorin Signaling in Neurons, Acute Phase Response Signaling, Ephrin Receptor Signaling, Chemokine Signaling, Leukocyte Extravasation Signaling, VEGF Signaling, Thrombin Signaling
8	AIRE, C15ORF63, Ca2+, CTSD, D-glucose, DAPK3, EEF2, ganglioside GM3, GBA2, GH1, GHR, GLYCOGEN PHOSPHORYLASE, GPR1, GRB2, heparin, HTT, LTF, MCHR2, MYL2, MYO3B, NISCH, P2RX7, PFN2, PKC ALPHA/BETA, PLA2G12A, PLA2G2D, Pld, PPA2, PPP1R12A, PPP1R1A, PPP3CA, PRF1, SELL, SLC7A9, SORBS1	16	11	Cell Signaling, Molecular Transport, Vitamin and Mineral Metabolism	Primary Immunodeficiency Signaling, Starch and Sucrose Metabolism, Atherosclerosis Signaling, Oxidative Phosphorylation, Eicosanoid Signaling, Cyanoamino Acid Metabolism, Stillbene, Coumanine and Lignin Biosynthesis, Phospholipid Degradation, Endothelin-1 Signaling, p38 MAPK signaling, MIF Regulation of Innate Immunity, CCR3 Signaling in Eosinophils, Arachidonic Acid Metabolism, Glycerophospholipid Metabolism, Linoleic Acid Metabolism
9	ARRB2, beta-estradiol, CCL5, CCL13, CCR5, Creatine Kinase, CSNK1A1L, EEF2, EIF2AK2, FOXO1, GAR1, HSP90AB1, KPNB1, KRI1, LAGE3, NHP2, OSGEP, OTUD5, PCNA, PPM1K, RAF1, RAN, RANGRF, RPLP0 (includes EG:6175), RPS6, SNRPE, SPN, SPRY2, SPRYD5, TACR1, TGFBR2, TNFRSF1B, TRAF3, XPO1, XPO5	16	11	Cell Death, Cell-mediated Immune Response, Cellular Movement	Leukocyte Extravasation Signaling
10	ALPHA AMYLASE, AMY1A, AMY1B, BMP8B, CD44, CSF1, DAG1, DNAH6, DYNLT1, EPO, FOXO1, FOXO3, FYN, glycogen, GRIN1, HCLS1, IL15, INPPL1, KCNH6, LCK, LMOD3, MIR9-1 (includes EG:407046), NMT1, oleic acid, PLCG2, Pld, PLIN5, RPS4Y2, Shc, SHC1, SHCBP1, TDRD1, VCAM1, VEGF	15	11	Cellular Development, Cell Cycle, Cell Death	Starch and Sucrose Metabolism, Factor Promoting Cardiogenesis in Vertebrates, Role of NANOG in Mammalian Embryonic Stem Cell Pluripotency, Role of Osteoblasts, Osteoclasts and Chondrocytes in Rheumatoid Arthritis, Basal Cell Carcinoma Signaling, BMP Signaling
11	ASB1, BEGAIN, C1ORF9, C8ORF45, CCDC85B, CTSD, DEFB103A, Eotaxin, EREG, ganglioside GD1a, ganglioside GD1b, ganglioside GM1, ganglioside GM2, ganglioside GM3, ganglioside GT1, GHR, IL32, KIAA0408, LHX4, MAFF, MAPK1, MIR202 (includes EG:387198), NDUFA5, NEUROG3, NRP1, PCBP2, PDE4C, PSAP, ROBO1, SEMA3D, SEMA3E, Sphk, TNF, ZFP36, ZNF337	14	12	Lipid Metabolism, Small Molecule Biochemistry, Cell Morphology	Purine Metabolism, Aminosugars Metabolism, Oxidative Phosphorylation, Lipid Antigen Presentation by CD1, Axonal Guidance Signaling, Ubiquinone Biosynthesis
12	ACTN1, BCAS2, C16ORF72, COPS2, CTTNBP2, CTTNBP2NL, DNTTIP2, EGR1, ESR1, ESR2, FAM40A, FAM40B, FAM63B, G alphai, JUN, MCC, MCM2, MIR122, MIR214 (includes EG:406996), MOBKL3, NR0B1, NR2F1, NRIP1, PPP2R1A, PTEN, retinoic acid, RNF122, RP6-213H19.1, SIKE1, STK24, STK25, STRN, STRN3, TRAF3IP3	9	7	Gene Expression, Cellular Development, Cellular Growth and Proliferation	Oxidative Phosphorylation

In contrast, the 172 genes and 12 networks identified from the second gene set included a much broader array of functional categories, ranging from DNA replication, recombination and repair, to cellular development and cell morphology, to cell-to-cell signaling and interaction. The genes identified were also implicated in a host of other functions including drug metabolism, endocrine system development and function, tumor morphology, molecular transport and even lipid metabolism (Table 2). What role each of these may play in the progression or recurrence of Dupuytren's pathology is as yet unclear.

An interesting feature of the Ingenuity analysis is that the data suggest the involvement of microRNAs (miRNAs) in the dysregulated processes leading to DC pathology. In examining the 120 genes concordantly expressed between PF- and CT-derived fibroblasts but significantly different in DC-fibroblasts, Ingenuity analysis identified two networks which showed direct interaction of three miRNAs, namely miRNA106A, miRNA17 and miRNA20B (Table 1). MicroRNA involvement is also suggested by analysis of the genes concordantly expressed between DC- and PF-fibroblasts but significantly different in CT. Three microRNAs are putatively implicated: miRNA202, miRNA122, miRNA214 (Table 2).

### Validation of the differential expression of select gene products by quantitative RT-PCR

**Figure 5:**
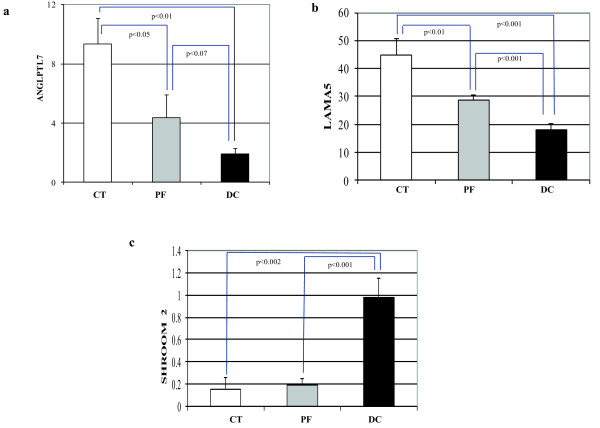
**Expression of ANGPTL7, LAMA5 and SHROOM2 as Determined by Microarray Analysis**. Differential expression of (a) ANGPTL7, (b) LAMA5 and (c) SHROOM 2 between CT-, PF- and DC-derived fibroblasts in the microarray. Signal intensities (in arbitrary units, after subtraction of background) for each gene product are shown. Statistical significance was derived using Significance Analysis of Microarrays program (SAM version 3.02). p < 0.05 was considered significant

**Figure 6:**
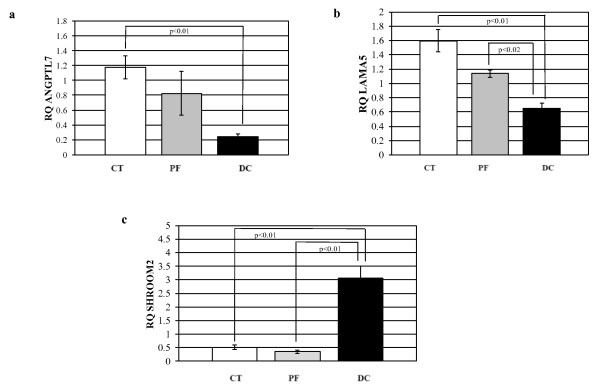
**Real time RT-PCR Quantitation of ANGPTL7, LAMA5 and SHROOM2 in CT-, PF-, and DC-derived cells**. Differential expression of (a) ANGPTL7, (b) LAMA5 and (c) SHROOM2 was directly confirmed by qRT-PCR in CT-, PF- and DC-derived fibroblasts. Values are mean ± SEM of two independent experiments performed in triplicate. GAPDH was used as an internal control. Statistical analyses were performed by Student's *t *test. Relative quantification of gene expression was calculated by comparing δ Ct values between CT-, PF- and DC- derived fibroblasts. p < 0.05 was considered significant

Quantitative real time RT-PCR was performed on the same RNA extracted for the microarray analysis on select genes to validate the microarray results (shown in Figure 5). The genes selected for such confirmation have not previously been reported to be differentially expressed in CT-, PF- or DC-derived fibroblasts. Angiopoietin-like7 mRNA (ANGPTL7) was significantly decreased in DC-derived fibroblasts compared to both PF- and CT- derived cells, amongst which no significant difference was seen (Figure 6a). LAMA5 mRNA was similarly least in DC-derived cells, but was also significantly less in PF- compared to DC-derived cells (Figure 6b). In contrast, Shroom2 message was dramatically increased in DC-derived fibroblasts compared to both CT- and PF-derived fibroblasts (Figure 6c). As can be seen by comparing the gene expression patterns in Figures 5 and 6, in each case quantitative RT-PCR findings closely matched the results from microarray analysis.

## Discussion

We previously reported significant differences in gene expression that distinguish fibroblasts obtained from Dupuytren's Contracture cords versus fibroblasts obtained from the normal fascia of patients undergoing surgical therapy for carpal tunnel syndrome [23]. The culture conditions in this study were modified from our previous report through the use of a type-1 collagen substrate as opposed to an uncoated standard plastic surface. Although the morphological phenotype of the fibroblasts appeared unaffected, many more differentially expressed genes (894 total) were detected in DC versus CT fibroblasts compared to the previous study (40 differentially expressed genes by Illumina BeadArray). Our results can be interpreted to indicate that palmar fascia fibroblasts are sensitive to their culture substrate at a molecular level despite minimal changes in morphological appearance and behavior. These findings can also be interpreted in light of our previous studies indicating that total cellular ß catenin levels in DC cells are altered by interactions with a type-1 collagen substrate [24]. Consistent with this, Ingenuity analysis on genes concordantly expressed between DC- and PF- but significantly different in CT-derived cells suggests the involvement of molecules in the Wnt/β-catenin signaling pathway. The gene encoding ß catenin, *CTNNB1*, was not amongst the dysregulated genes identified in this analysis, consistent with the primary regulation of cellular ß catenin levels through post-translational mechanisms rather than transcriptional induction [27]. Viewed in combination, our findings suggest that the sensitivity of fibroblasts derived from the palmar fascia of DC patients to their immediate environment is evident at both transcriptomic and proteomic levels. These findings may have profound implications for *in vitro *culture models designed to replicate DC *in vivo *and highlight the potentially important effects of culture substrates on cellular phenotypes.

There are at least two non-exclusive, potential explanations for the marked differences in gene expression between primary palmar fascia fibroblasts grown on plastic tissue culture trays and those grown on a type-1 collagen substrate. The collagen concentration used for these analyses was 1.9 mg/ml, yielding a relatively soft substrate that can be readily contracted by differentiated myofibroblasts in fibroblast populated collagen lattice assays [24]. A stiff and non-deformable substrate, such as tissue culture plastic, may promote myofibroblast differentiation in a similar manner to that induced by increased tissue density [28]. This differentiation may be due, at least in part, to the activation of latent TGF-ß1 secreted by these cells into their extra-cellular matrix [29]. Under these culture conditions, the differences in gene expression in primary fibroblasts from different tissues may be masked by the changes in gene expression induced during myofibroblast differentiation. In contrast, a soft and readily deformable type-1 collagen substrate may have prevented substrate induced myofibroblast differentiation and allowed for more sensitive detection of the original changes in gene expression that more closely reflect the tissues of origin. Additionally, collagens have been reported to induce a "proliferation permissive" signal through a beta1 integrin-mediated PI3 Kinase/Akt pathway in fibroblasts in other systems [30]. Interactions with type-1 collagen may, therefore, activate gene transcription through this, and possibly other, pathways in CT, PF and DC cells and induce the differential expression of genes that are not activated in the absence of collagen. Thus, culturing primary fibroblasts on a type-1 collagen substrate could induce any or all of these effects and explain the marked increase in differentially expressed genes identified in our analyses.

It is notable that each different cell group (DC vs. PF vs. CT) displays a remarkable internal consistency. Six unrelated individuals are compared in each cell group and were found to have highly similar transcriptomic profiles, as evidenced by both hierarchical clustering and concordance mapping. This lends confidence to interpretation of our data as a meaningful representation of the actual differences between the tissue and disease states examined. The most compelling observation is that palmar fascia fibroblasts from phenotypically normal tissue in DC patients closely resemble (but, importantly, are not transcriptomically identical to) cells derived from clearly phenotypically abnormal tissue. As a corollary, both fibroblasts from diseased DC cords and uninvolved DC fascia are markedly transcriptomically dissimilar to fibroblasts from carpal tunnel fascia. This observation is strongly suggestive that a major contributor to the tendency for DC recurrence may be a genetic predisposition towards disease of phenotypically normal palmar fascia in DC patients. Such an interpretation would be consistent with previous reports that DC has a genetic basis {eg. Hu et al., 2005 [3]}. Our findings may also reflect on recent reports suggesting that DC cells may be derived from mesenchymal stem cells (MSCs) in perinodular fat and skin [31]. While our analyses do not rule out the possibility that MSCs from these tissues contribute to (or perhaps initiate) disease progression or recurrence, the marked similarity between the transcriptomes of DC and PF cells and their relative dissimilarity to CT cells strongly implies that the vast majority of cells in DC cord tissue are likely to be derived from PF cells.

These observations make it possible to consider the progression and recurrence of DC in a model based on principles first elucidated in tumor biology, the so-called "two-hit hypothesis" [32] first described by Nordling in 1953 [33]. In such a model we hypothesize that an inherited allelic defect (or possibly a group of allelic defects) constituting a first "hit" alters palmar fascial physiology in DC patients, but not sufficiently so as to lead to the DC nodule/cord phenotype. Progression to frank and active disease would require a second somatic mutation (the second "hit") in the remaining allele in the affected tissue type. Such a model would account for the observation that DC is a heritable disorder, usually presenting later in life (with correspondingly greater chances of having accrued the second requisite mutation), but with widely varying penetrance. Given that both PF and DC cells display hundreds of genes differentially expressed at the mRNA level compared to control CT cells, the putative etiological locus may be involved in transcriptional regulation. Of course, it remains possible that DC is a heterogeneous disorder with a similar phenotype arising from multiple disparate underlying genetic loci, nor does such a model eliminate the possibility of *de novo *sporadic cases of DC.

These findings also have potential implications for *in vitro *studies designed to dissect the molecular mechanisms of DC. If patients with a genetic predisposition to develop DC have inherited PF cells that exhibit profound differences in gene expression to normal palmar fascia (CT) cells, then PF cells represent the most clinically relevant controls for testing treatments designed to prevent DC progression. In contrast, normal palmar fascia cells, such as CT cells, are valuable for analyses designed to identify the molecular characteristics that distinguish DC cells from normal cells. Thus, while identifying differences in signaling pathway activation between DC and CT cells [34] are useful analyses to characterize differences from normality, it is clearly essential that such pathways are also assessed in PF cells before they are considered potential therapeutic targets. A study by Pavelic et al., [35] employing a proteomic approach has identified several proteins interacting in various signaling pathways in DC tissues when compared to unaffected palmar fascia from DC patients. This identification of protein-protein interaction potentially opens new therapeutic targets to reduce the recurrence of DC. Optimally, comparisons of DC cells to both CT and PF cells are likely to provide different, but equally valuable, insights into DC progression and recurrence that will allow researchers to distinguish and identify molecular or other therapies that target DC, but not PF or CT, cells.

The remarkable internal consistency between samples in the microarray was also reflected in the findings obtained from real time RT-PCR assays used to validate the microarray results. In seeking potentially novel targets to limit the progression of DC, we chose to directly examine three select genes that have not been previously implicated in the progression or recurrence of DC. Human ANGPTL7 had been characterized as a potent target gene of the WNT/β-catenin signaling pathway and currently is a pharmacogenomics target in the fields of oncology and regenerative medicine [36]. Studies by Kuchtey et al., (2008) [37] and subsequent studies by Comes et al., (2010) [38] showed that ANGPTL7 has the potential to alter extracellular matrix formation, a finding that may also be relevant to DC. We find that in DC-derived fibroblasts ANGPTL7 mRNA is substantially decreased in comparison to CT- and PF-derived fibroblasts (Figure 5a and 6a). The functional importance of ANGPTL7 in the biology of DC is unknown and further studies are required to understand its role in DC progression.

We also directly examined the extracellular matrix protein laminin isoform, laminin alpha5 (LAMA5). Laminin isoforms have been shown to play crucial roles in modulating cell adhesion, proliferation, differentiation, and migration in normal and pathological states by interacting with other extracellular matrix components [39,40]. In both microarray and by quantitative RT-PCR we found that LAMA5 mRNA expression was specifically increased in CT- and PF-derived cells compared to DC-derived fibroblasts (Figure 5b and 6b). A previous study by Kosmehl et al., (1995) [41] showed higher expression of laminin A, as well as M, B1, B2 and S chains, in Dupuytren nodular tissues, but LAMA5 expression in cord tissue is unknown. Further dissection of the functional significance LAMA5 might provide valuable insights for manipulation to limit the progression of DC.

In contrast to ANGPTL7 and LAMA5, direct examination of Shroom2 message showed a marked increase in DC-derived cells (~ 5-6- fold) compared to CT- or PF-derived fibroblasts, again mirroring the findings from the microarray data (Figures 5c &6c). Shroom family proteins are reported to interact with actin and actin polymerization is required for their function [42,43]. Moreover, members of the Shroom family (mainly Shrooms 1, 2 and 3) cause accumulation of γ-tubulin, a microtubule nucleating protein, at the apical surface of epithelial cells [44]. Based on these and other findings it has been suggested that Shroom genes function in the regulated control of cytoskeletal molecules and thereby aid in cell morphogenesis. We have found that interference with actin pathways can affect Dupuytren's fibroblast contractility (Satish et al., manuscript in preparation) and enhanced levels of Shroom 2 may, be one of the factors that alter actin-mediated cell contraction in DC, potentially identifying it as an attractive target through which to inhibit disease progression or recurrence.

Ingenuity analysis of our differentially expressed gene sets has identified multiple microRNAs that may play a role in DC pathophysiology, although direct experimental evidence is as yet lacking. Recently Mosakhani et al., [45] for the first time examined expression of microRNAs in affected DC tissues, comparing them to normal fibroblasts in culture and normal fascia obtained from hand trauma and carpal tunnel patients. Curiously, although they identified dozens of differentially expressed microRNAs, none of the microRNAs suggested by our Ingenuity analysis appeared in their screen. This may be due to the disparate natures of the sample sources (ie. tissues containing a mixture of cells of mesenchymal, endothelial and other lineages versus primary fibroblasts in culture) or other reasons, such as the absence of mechanical stress to activate mechanoreceptors, or additional extra-cellular matrix components and matricellular molecules that may, directly or indirectly, regulate microRNA transcription. We anticipate that additional analyses incorporating some or all of these potential effectors of DC development *in vivo *will be required to establish the full relevance of the differential gene expression patterns we report here.

## Conclusions

These data show that the transcriptomic profiles of DC-disease fibroblasts and fibroblasts from unaffected palmar fascia in DC patients are highly similar, and differ significantly from the transcriptomic profiles of fibroblasts from the palmar fascia of patients undergoing carpal tunnel release. These observations are further evidence of an inherent molecular pathophysiology in DC disease fibroblasts that may help to explain the progression of the disease and its tendency to recurrence.

## Competing interests

The authors declare that they have no competing interests.

## Authors' contributions

LS, SK, DO'G and BSG conceived and designed the experiments. LV, AN, CR, LS and SJ performed the experiments. PHG performed the real time RT-PCR experiments. Analysis tools were contributed by WAL and JMKB. Data analyses were performed by WAL, JMKB, LS, SK, DO'G, BSG. Cell cultures/materials were contributed by DO'G, BSG, MEB, GDE. The manuscript was drafted by SK, LS and WAL. The manuscript was critically reviewed by MEB, LV, AN, CR, DO'G, BSG, GDE. All authors have read and approved the final manuscript.

## Pre-publication history

The pre-publication history for this paper can be accessed here:

http://www.biomedcentral.com/1755-8794/5/15/prepub

## Supplementary Material

Additional file 1 **Differences in individual gene expression levels identified through SAM analysis**. Click here for file
